# The Association of Serum L-Carnitine Concentrations with the Risk of Cancer in Chinese Adults with Hypertension

**DOI:** 10.3390/nu14234999

**Published:** 2022-11-24

**Authors:** Tong Liu, Chenan Liu, Xiaomeng Wang, Yaping Wei, Shuqun Li, Yun Song, Ping Chen, Lishun Liu, Binyan Wang, Hanping Shi

**Affiliations:** 1 Key Laboratory of Cancer FSMP for State Market Regulation, Department of Gastrointestinal Surgery/Clinical Nutrition, Beijing International Science and Technology Cooperation Base for Cancer Metabolism and Nutrition, Capital Medical University Affiliated Beijing Shijitan Hospital, Beijing 100038, China; 2 Department of Education, Capital Medical University Affiliated Beijing Shijitan Hospital, Beijing 100038, China; 3 Key Laboratory of Precision Nutrition and Food Quality, Ministry of Education, Department of Nutrition and Health, College of Food Sciences and Nutritional Engineering, China Agricultural University, Beijing 100083, China; 4 Shenzhen Evergreen Medical Institute, Shenzhen 518000, China

**Keywords:** L-carnitine, incident cancer, hypertension, TMAO

## Abstract

Background: The effect of serum L-carnitine (LC) concentrations on cancer risk remains unclear. This study aims to explore the association between serum LC and the risk of incident cancer. Methods: This is a case-control study, including 574 patients with incident cancer and 574 controls matched in a 1:1 ratio by age, sex, and residence, nested within the China H-Type Hypertension Registry Study (CHHRS). Conditional logistic regression analysis was used to assess the association of serum LC and incident cancer risk. Results: When LC was assessed as quartiles, compared with patients with low LC (Q1), patients in the highest quartile (Q4) had a 33% (OR = 0.67, 95% CI: 0.46 to 0.99), 52% (OR = 0.48, 95% CI: 0.23 to 0.99), and 39% (OR = 0.61, 95% CI: 0.38 to 0.99) decreased risk of overall, digestive system, and non-digestive system cancer in the adjusted models, respectively. In subgroup analyses, an inverse association of LC with cancer risk was observed in individuals who were overweight (obese), who never drink, who never smoke, and who were female. In the mediation analysis, serum trimethylamine-*N*-oxide (TMAO) concentrations did not mediate the reversed association of LC with cancer risk. Conclusions: This study showed that serum LC concentrations had a protective impact on overall, digestive system, and non-digestive system cancer risk.

## 1. Introduction

The cancer burden is rising worldwide. Cancer has the greatest impact and the fastest rate of incumbrance in low- and middle-income nations, many of which are already struggling to cope with the present burden. GLOBOCAN 2020 estimated that there were 19.3 million incident cancer cases and 10.0 million cancer deaths globally in 2020 [1]. Female breast cancer is the most commonly diagnosed cancer type (11.7%), followed by lung cancer (11.4%), colorectal cancer (10.0%), prostate cancer (7.3%), and stomach cancer (5.6%). Several established risk factors have been identified for all cancers, including tobacco smoking, unhealthy diet, obesity, and infectious agents [2,3,4]. However, numerous carcinogenic factors still need to be explored through further research. Determining the causes of cancer will influence potential screening and prevention approaches.

L-carnitine (LC), a naturally occurring quaternary ammonium molecule, is mostly obtained from animal-based foods (75%) and, to a lesser extent, is endogenously produced in the liver and kidney (25%) [5]. The primary function of LC is to transport long-chain fatty acids into the mitochondrial matrix for energy conversion via the β-oxidation process [6]. Additionally, LC controls pyruvate dehydrogenase activity [6] and maintains the proper protein balance in skeletal muscle [7]. Daily intake of LC has been shown to improve performance and energy expenditure [8], protect muscles from atrophy [9], reduce body weight [10], and increase the amount of protein retention [11]. LC may reduce the risk of cancer due to its weight-lowering effect. However, oral LC supplementation increases the levels of trimethylamine-*N*-oxide (TMAO) [12], which, as a pro-atherogenic factor, has been indicated to increase the risk of cardiovascular disease (CVD) [13], chronic kidney disease (CKD) [14], and even cancer [15]. Therefore, it can be speculated that LC may elevate the risk of cancer incidence due to the close relationship between LC and TMAO.

The effect of serum LC concentration on cancer risk remains unclear. In the current study, we performed a nested, case-control study to explore the association between serum LC levels and the risk of cancer incidence, by drawing data from a community-based population in China.

## 2. Methods

### 2.1. Study Population and Design

The current study is a subset of the China H-Type Hypertension Registry Study (CHHRS; URL: http://www.chictr.org.cn/showprojen.aspx?proj=28262; unique identifier: ChiCTR1800017274), a community-based, prospective, observational, real-world registry study that is currently being conducted primarily in Lianyungang City, Jiangsu Province, China. The goals of this study are to (1) create a national hypertension patient registration system; (2) investigate the prevalence and treatment of H-type hypertension in China, as well as the factors that influence its prognosis; and (3) develop a risk prediction model for cardiovascular, cerebrovascular, and renal diseases. Participants over the age of 18 with hypertension—defined as seated systolic blood pressure (SBP) ≥ 140 mmHg and/or seated diastolic blood pressure (DBP) ≥ 90 mmHg at the screening visit—were eligible to participate. Participants with impaired psychiatric or neurological conditions were excluded. The current study is divided into two phases: a recruiting (screening) period and a three-year observational follow-up period. Routine follow-ups were scheduled every three months to collect measurement data, to record blood pressure and medication usage, and to study outcome events including CVD, cancer, and all-cause mortality.

### 2.2. Nested Case-Control Study

**Figure 1:**
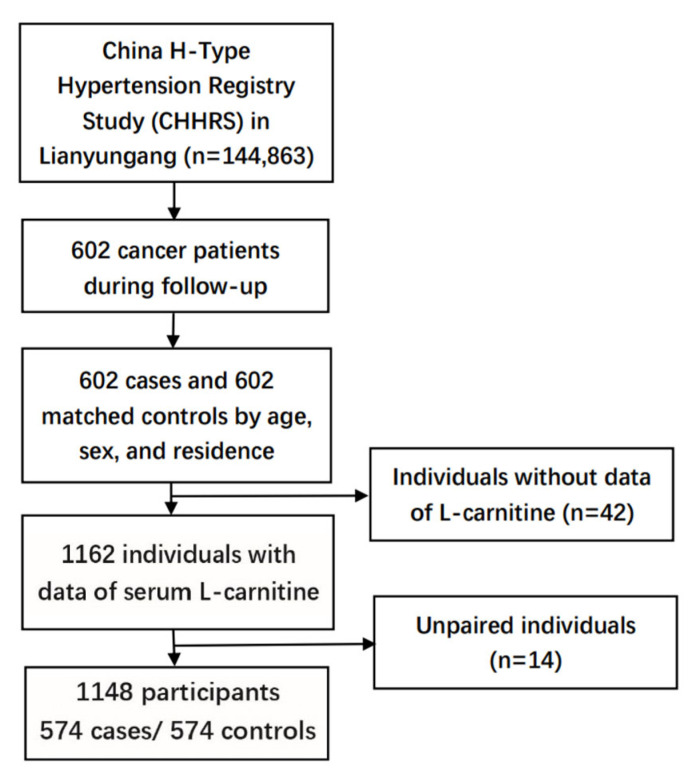
Flowchart of study participants in the nested case-control study within the CHHRS.

During the CHHRS follow-up, 602 cancer cases occurred in Lianyungang District among 144,863 participants. A nested case-control study comprising 602 newly diagnosed cancer cases and 602 matched controls from the same district was conducted. Those individuals who had not developed cancer during the follow-up were chosen as controls, and they were matched by age (±1 year), sex, and residence, in a 1:1 ratio. After excluding patients with missing LC measurements (*n* = 42) and unpaired individuals (*n* = 14), a total of 1148 participants (574 cancer cases vs. 574 matched controls) were included in the final analysis (Figure 1), with 213 digestive system cancer and 361 non-digestive system cancer cases.

The CHHRS and the present study were approved by the Ethics Committee of the Institute of Biomedicine, Anhui Medical University, Hefei, China. All participants or a family member representative signed the informed consent after the study protocol was thoroughly explained to them.

### 2.3. Outcome Ascertainment

Cancer cases were identified based on certain clinical criteria or positive histopathologic data from hospitals where patients had received cancer therapy from 2016 to 2019. Potential cases were further assessed by two oncologists when pathological data were lacking. Cancer cases were identified only when the two professionals made the same cancer diagnosis and were coded using the International Classification of Diseases, Tenth Revision (ICD-10).

### 2.4. Exposure and Potential Confounders

Overnight fasting (≥8 h) venous blood samples were collected using vacuum tubes containing EDTA. The levels of TMAO and L-carnitine were determined using stable isotope dilution liquid chromatography–tandem mass spectrometry (6460 Series Triple Quadrupole LC/MS; Agilent, Santa Clara, CA, USA). Serum levels of fasting lipids including alanine aminotransferase (ALT), albumin (ALB), triglycerides (TG), total cholesterol (TC), high-density lipoprotein cholesterol (HDL-C), fasting blood glucose (FBG), uric acid (UA), and creatinine obtained at baseline were measured using automatic clinical analyzers (Beckman Coulter) at the Shenzhen Tailored Medical Laboratory.

A standard questionnaire was used to collect information on the socioeconomic status, lifestyle behaviors, and medical history of participants and their family members. Body measurements were conducted for all participants by trained medical staff.

### 2.5. Statistical Analysis

A two-tailed *p* < 0.05 was considered statistically significant in all analyses. All statistical computations were performed using SAS software version 9.4 and R software version 3.4.3 (https://www.r-project.org, accessed on 1 January 2022.).

Normally distributed variables were presented as means ± SD and compared using paired *t*-tests. Non-normally distributed variables were presented as medians ± IQR and analyzed using nonparametric Kruskal–Wallis tests. Categorical variables were presented as *n* (%) and compared using χ2 tests or Fisher’s exact tests. Restricted cubic spline regression (RCS) was used to calculate the dose-response association between LC and cancer risk. Conditional logistic regression models were used to assess the association between serum L-carnitine (continuous and categorical variables) and the risk of incident cancer. Patients were categorized into four subgroups according to quartiles of serum LC at baseline: quartile 1 (Q1, <4.72 μg/mL), quartile 2 (Q2, 4.72–5.78 μg/mL), quartile 3 (Q3, 5.79–6.84 μg/mL), and quartile 4 (Q4, ≥6.85 μg/mL). Multivariate analyses were conducted by adjusting for ALT, ALB, body mass index (BMI), smoking status, alcohol drinking, SBP, TC, TG, UA, HDL-C, glucose, creatinine, sleep quality, meat consumption frequency, type of meat consumed, TMAO, oral antihypertensive medication, and family history of cancer. Participants were then divided into 2 groups by the median of LC to further validate the results. Finally, stratified analyses and interaction tests were conducted to investigate the modifying effect of relevant factors on the relationship of LC levels as a binary variable (median) with cancer. To better explore the relationship between meat consumption and the levels of LC and TMAO, we compared the overall differences in levels of TMAO and LC among meat intake frequency groups by using one-way analysis of variance (ANOVA), and SNK-q test was used for the comparison within each group.

The effect of serum LC levels on the risk of the digestive system and non-digestive system cancers according to quartiles of serum LC at baseline were further explored. The quartile cut-off values for digestive system cancer were 4.88, 5.95, and 7.00 µg/mL, respectively, and the quartile cut-off values for non-digestive system cancer were 4.62, 5.73, and 6.69 µg/mL, respectively. Due to the close relationship between LC and TMAO, the association of TMAO with incident cancer risk was additionally investigated using conditional logistic regression models.

TMAO has been demonstrated to be closely related with the levels of LC. In addition, though the results are inconsistent, the close relationship between TMAO and cancer risk has been explored previously. To further explore whether the effect of serum LC concentrations on the occurrence of cancer is mediated by levels of TMAO, the CAUSALMED procedure was utilized by using the variance–covariance matrix and the maximum likelihood method. This procedure calculated the total effect (the sum of the direct and indirect effects), the direct effect (the effect without the mediator’s influence), and the indirect effect (the independent variable’s influence on the mediator multiplied by the mediator’s influence on the outcome).

## 3. Results

### 3.1. Baseline Characteristics of the Participants

We included 574 cancer cases in the current study, comprising 213 patients with digestive system cancer, and 361 patients with non-digestive system cancer. Table 1 summarizes the baseline characteristics of cancer patients and matched controls. Compared with matched controls, cancer patients had decreased levels of ALB and LC, were more likely to smoke and have poor sleep quality, and had a higher prevalence of a history of coronary heart disease (CHD) and stroke. Notably, cancer patients were more likely to consume fatty meat rather than lean mean compared to the control group. No differences were found in age, sex, BMI, blood pressure, ALT, TG, TC, UA, HDL-C, FBG, creatinine, TMAO, marital status (married), educational background (high school or above), current drinker, history of CKD, family history of cancer, antihypertensive medication usage, and meat intake frequency between the cancer patients and controls (all *p*-values for differences > 0.05).

### 3.2. Association of LC with the Risk of Overall, Digestive, and Non-Digestive System Cancer

**Figure 2:**
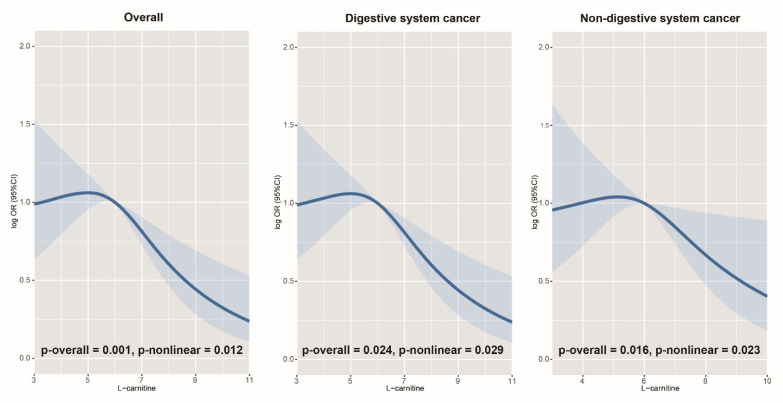
Association between serum LC concentrations and cancer risk using RCS.

The median follow-up period for the current study was 3.02 (1.92, 3.40) years. The association between LC concentrations and incident cancer risk is shown in Figure 2. We found an inverse and nonlinear relationship between LC concentrations and the risk of overall (*p*-overall = 0.001, *p*-nonlinear = 0.012), digestive system (*p*-overall = 0.024, *p*-nonlinear = 0.029), and non-digestive system cancer (*p*-overall = 0.016, *p*-nonlinear = 0.023). Table 2 and Table 3 present the ORs (95% CI) for the association of LC with the risk of overall, digestive system, and non-digestive system cancer. Serum LC (per unit increment) significantly lowered the risk of overall cancer (OR = 0.86, 95% CI: 0.79 to 0.94), digestive system cancer (OR = 0.82, 95% CI: 0.69 to 0.97), and non-digestive system cancer (OR = 0.87, 95% CI: 0.78 to 0.98) in the multivariate analysis. Compared with patients with low LC (Q1), patients in the highest quartile (Q4) had a 33% (OR = 0.67, 95% CI: 0.46 to 0.99), 52% (OR = 0.48, 95% CI: 0.23 to 0.99), and 39% (OR = 0.61, 95% CI: 0.38 to 0.99) decreased risk of overall, digestive system, and non-digestive system cancer in the adjusted models, respectively. Patients with higher concentrations (≥median) of LC also exhibited a decreased risk of overall (OR = 0.75, 95% CI: 0.57 to 0.99) and digestive system cancer (OR = 0.57, 95% CI: 0.34 to 0.97) in the adjusted models compared to those with lower concentrations (<median).

**Figure 3:**
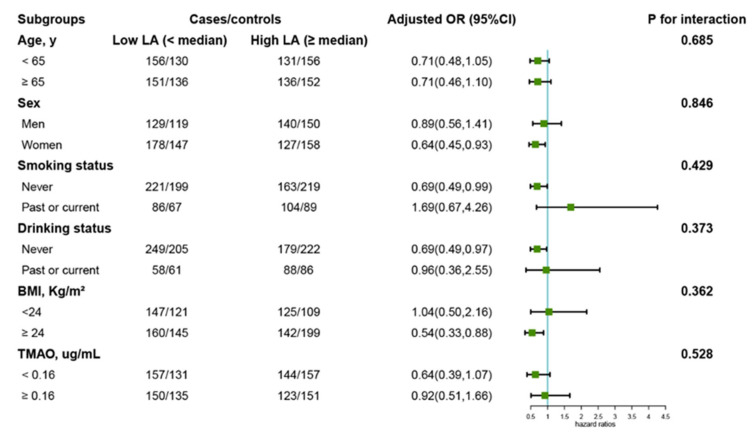
Stratified analysis of the association of serum LC with the risk of overall cancer. Note: Models were adjusted for ALT, ALB, BMI, smoking status, alcohol drinking, SBP, TC, TG, UA, HDL-C, glucose, creatinine, sleep quality, meat consumption frequency, type of meat consumed, TMAO, oral antihypertensive medication usage, and family history of cancer, except for the stratified factors.

Figure 3 illustrates the subgroup analysis of the association of serum LC (low vs. high divided by the median of LC) with overall cancer risk. Age (<65 (median) vs. ≥65 y), sex, smoking (never vs. past or current) and drinking (never vs. past or current) status, BMI (normal <24 vs. overweight (obese) ≥24 kg/m^2^), and TMAO (<0.16 (median) vs. ≥0.16 µg/mL) did not modify the effect of LC on the occurrence of overall cancer risk (all *p* for interaction < 0.05). An inverse association of LC with cancer risk was observed in individuals with higher BMI (≥24 kg/m^2^), never drinkers, never smokers, and females. We also explored the effect of serum TMAO levels on the occurrence of overall cancer (Table 4). In the current study, participants with elevated levels of TMAO (Q3, 0.16–0.25 µg/mL) had a 43% decreased risk of overall cancer in the adjusted model (OR = 0.57, 95% CI: 0.37 to 0.90).

**Figure 4:**
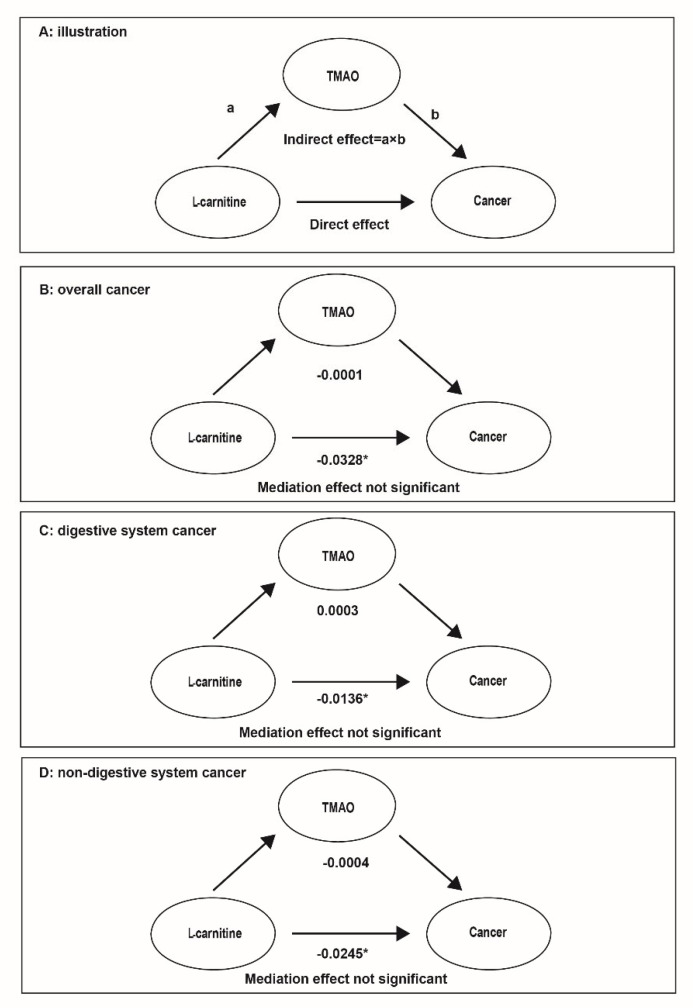
The mediation effect between LC, TMAO, and cancer risk. Note: Models were adjusted for ALT, ALB, BMI, smoking status, alcohol drinking, SBP, TC, TG, UA, HDL-C, glucose, creatinine, sleep quality, meat consumption frequency, type of meat consumed, oral antihypertensive medication usage, and family history of cancer, except for the stratified factors. * Values were statistically significant.

In the mediation analysis, we found a significant direct effect of LC concentrations on occurrence of overall, digestive system, and non-digestive system cancer. However, the indirect effect (the effect of the LC on the TMAO multiplied by the effect of the TMAO on the cancer risk) and the mediation effect were not significant. The serum TMAO concentrations did not mediate the reversed association of LC with the risk of overall, digestive system, and non-digestive system cancer (Figure 4).

## 4. Discussion

This population-based, prospective, case-control study, nested within the CHHRS, found that individuals with hypertension with greater serum LC levels had a lower risk of overall cancer. Significant associations were similarly observed for digestive system and non-digestive system cancer. Similar results were also identified among participants with higher BMI, never drinkers, never smokers, and females. In addition, the reversed association of LC with cancer risk was not mediated by TMAO concentrations.

In the current study, we firstly found a non-linear and reversed association between serum LC and cancer risk. The risk of cancer is initially elevated and then significantly decreased with the increase in the levels of LC. To date, only one existing study has explored the effect of serum LC on the occurrence of cancer. A nested case-control study of 644 colorectal cancer cases and 644 controls failed to find a positive association between serum carnitine and colorectal cancer risk in male smokers in the Alpha-Tocopherol, Beta-Carotene Cancer Prevention (ATBC) Study [16]. In addition, an experimental study revealed that Acetyl-L-carnitine (ALCAR) decreased prostate cancer cell proliferation, adhesion, migration, and invasion both in vitro and in vivo, suggesting a new potential for ALCA as a “repurposed agent” for cancer prevention and interception [17]. In another study by Baci D. et al., it was found that ALCAR may be an excellent option for prostate cancer angioprevention because it possesses anti-angiogenic and anti-inflammatory effects [18]. Wenzel U. et al. discovered that a combination of palmitoyl-carnitine and L-carnitine potently triggered apoptosis in HT-29 human colon cancer cells as a result of increased fatty acid oxidation [19].

L-carnitine insufficiency is commonly found among cancer patients, which was also the case in the current study. According to previous studies, up to 80% of patients with advanced malignant tumors have an irreparable lack of L-carnitine, leading to symptoms such as fatigue, malnutrition, and depression in cancer patients [20,21,22]. There are several reasons that help to explain the LC deficiency found in cancer patients: (1) inadequate diet (e.g., deficiencies in iron, vitamin C, and L-methionine); (2) disruption of L-carnitine production by anthracyclines; (3) increase in renal L-carnitine excretion by cisplatin and ifosfamide; (4) competition with anthracyclines for the carnitine transporter OCTN2, which is required for L-carnitine transfer into cells.

Despite the limited data on the association of serum LC concentrations with cancer risk, L-carnitine treatment is also used to prevent the neuro- or cardiotoxicity linked to chemotherapy as well as fatigue in the setting of anticancer medication. Several studies have demonstrated that adjuvant treatment of L-carnitine at dosages ranging from 2 g to 6 g per day may prevent weight loss and/or weakness and fatigue in cancer patients [23,24]. Based on the findings of a prospective, uncontrolled investigation including 12 patients with advanced malignancies [25] and one randomized controlled trial [21], there is evidence that L-carnitine has favorable effects on parameters associated with cancer anorexia–cachexia syndrome (CACS). In addition, patients suffering from cachexia may benefit from a combination of L-carnitine and omega-3 fatty acids. Due to the anti-inflammatory properties of omega-3 fatty acids, recent investigations have considered their function in cancer prevention, cancer cachexia treatment, and anti-tumor therapy improvement [26,27].

In the current study, we found a negative association between serum TMAO and subsequent cancer risk. Previous research has revealed that TMAO protects against carcinogenesis by repairing folding errors in mutant proteins [28,29]. However, contrary to our findings, both epidemiological and experimental studies have found a positive association of TMAO levels with cancer risk [15,30]. Whether serum TMAO levels act as a lurking variable or a causative factor needs to be further explored in future studies. In addition, previous studies found meat (particularly red meat) consumption significantly elevated the levels of LC and TMAO [12,31]. In the current study, we found that serum L-carnitine levels increased as the frequency of weekly meat intake increased (Figure S1); participants who consumed meat > 3 times/week had significantly higher levels of LC than participants who seldom consumed meat (6.09 μg/mL vs. 5.73 μg/mL, *p* < 0.05). However, no significant differences were found for the levels of TMAO among participants with different meat-intake frequencies (Figure S1).

Several underlying mechanisms may help explain the reversed association between serum LC concentrations and subsequent cancer risk. First, as a key to mitochondrial ß-oxidation to generate energy, LC was found to significantly reduce body weight, BMI, and fat mass, especially among overweight (obese) adults [32]. Excess body weight is associated with increased cancer risk through biological mechanisms including abnormalities in the IGF-I system and signaling, insulin resistance, chronic low-grade inflammation and oxidative stress, altered pathophysiology of adipokines, ectopic fat deposition factors, etc. [33]. Second, LC alleviates inflammation by suppressing the release of crucial pro-inflammatory and pro-angiogenic factors [34,35]. Both intrinsic and extrinsic inflammation may lead to immunosuppression, which creates a favorable environment for the growth of tumors. Third, in vitro, LC can decrease tumor development markers such as PC-3, LNCaP, DU145, and BPH cell adhesion, migration, and invasion [17]. Fourth, LC affects endothelial cells, disrupting the VEGF/VEGFR2 and CXCR4/CXCL12 axes and lowering angiogenesis in vitro.

The main strength of this current study is that it provides a unique perspective on the possible protective effects of serum LC concentrations on the occurrence of incident cancer risk among a Chinese population with hypertension. In addition, the prospective study design eliminates recall bias and is more suitable for time-to-event analysis. Moreover, the broader assessment of confounders including the type of meat consumed and serum TMAO concentrations further validates the robustness of the main findings.

Several limitations should also be noted for the current study. First, participants in the current study were all patients with hypertension with a mean age of 62 years; whether our findings are directly relevant to younger patients with hypertension or the general population requires additional examination. Second, our results were based on a single measurement of LC levels at baseline, which may yield a certain degree of variability during the follow-up, and, therefore, we were unable to examine how variations in LC concentrations over time affect the occurrence of cancer. Third, although we investigated the effect of serum LC levels on digestive system and non-digestive system cancers, the effect of LC on site-specific cancers was difficult to assess due to the relatively small sample size. Last, this study did not contain detailed data on other cancer-associated factors including infectious agents, physical activity, etc., which hindered us from assessing confounding factors more precisely.

## 5. Conclusions

This study showed that serum LC concentrations had a protective impact on overall, digestive system, and non-digestive system cancer risk. If further verified, our study’s findings could provide a straightforward, safe, new path for cancer prevention given the heavy burden and fatalities from cancer in China and throughout the world.

## Figures and Tables

**Table 1:** Baseline characteristics of the cancer cases and matched controls.

Variables	Controls (*n* = 574)	Cases (*n* = 574)	*p*-Value
Age, year	64.78 ± 9.53	64.78 ± 9.53	0.995
Men, *n* (%)	269 (46.86)	269 (46.86)	1.000
BMI, kg/m^2^	24.89 ± 3.33	24.55 ± 3.95	0.114
Baseline SBP, mmHg	139.46 ± 18.59	138.60 ± 18.77	0.438
Baseline DBP, mmHg	86.00 ± 13.64	85.84 ± 14.53	0.845
ALT, U/L	15.0 (12.0, 21.0)	15.0 (11.0, 21.0)	0.564
ALB, g/L	44.61 ± 2.42	44.10 ± 2.94	0.002
TG, mmol/L	1.41 (0.92, 2.29)	1.30 (0.88, 2.14)	0.258
TC, mmol/L	5.60 ± 1.08	5.54 ± 1.04	0.322
UA, umol/L	305.0 (259.0, 356.0)	305.0 (255.0, 358.0)	0.863
HDL-C, mmol/L	1.15 ± 0.25	1.15 ± 0.26	0.795
FBG, mmol/L	6.36 ± 1.75	6.29 ± 1.47	0.493
Creatinine, umol/L	53.0 (46.0, 62.0)	53.0 (47.0, 62.0)	0.756
TMAO, μg/mL	0.16 (0.10,0.25)	0.16 (0.09,0.26)	0.612
L-carnitine, μg/mL	6.07 ± 1.81	5.65 ± 1.49	<0.001
Marital status, (married, *n* (%))	495 (86.24)	487 (84.84)	0.502
High school or above, *n* (%)	32 (5.57)	23 (4.01)	0.214
Current smoker, *n* (%)	109 (18.99)	148 (25.78)	0.022
Current drinker, *n* (%)	120 (20.91)	110 (19.16)	0.423
History of CKD, *n* (%)	4 (0.70)	6 (1.05)	0.525
History of CHD, *n* (%)	0 (0)	39 (6.79)	<0.001 *
History of stroke, *n* (%)	0 (0)	70 (12.20)	<0.001 *
Family history of cancer, *n* (%)	18 (3.14)	26 (4.53)	0.170
Poor sleep quality, *n* (%)	66 (11.50)	97 (16.90)	0.015
Antihypertensive drug usage, *n* (%)	193 (33.62)	186 (32.40)	0.660
Meat intake per week, *n* (%)			0.104
Seldom	265 (46.17)	302 (52.61)	
1–2 times	201 (35.02)	181 (31.53)	
≥3 times	108 (18.81)	91 (15.95)	
Type of meat consumed, *n* (%)			0.028
None	136 (23.69)	166 (28.92)	
Lean meat	309 (53.83)	281 (48.95)	
Mixed lean and fatty	92 (16.03)	74 (12.89)	
Fatty meat	37 (6.45)	53 (9.23)	

Note: BMI: body mass index; SBP: systolic blood pressure; DBP: diastolic blood pressure; ALT: alanine aminotransferase; ALB: albumin; TG: triglycerides; TC: total cholesterol; UA: uric acid; HDL-C: high-density lipoprotein cholesterol; FBG: fasting blood glucose; TMAO: trimethylamine-*N*-oxide; CKD: chronic kidney disease; CHD: coronary heart disease. * Compared by Fisher’s exact test.

**Table 2:** The association of L-carnitine with overall cancer risk.

L-Carnitine (μg/mL)	Cases/Controls (Ratio 1:1)	Crude Model		Adjusted Model	
		OR (95% CI)	*p*-Value	OR (95% CI)	*p*-Value
Per 1-unit increment	574/574	0.87 (0.81, 0.93)	<0.001	0.86 (0.79, 0.94)	0.001
Quartiles					
Q1 (<4.72)	160/130	Ref.		Ref.	
Q2 (4.72–5.78)	147/136	0.90 (0.63, 1.27)	0.543	0.83 (0.56, 1.24)	0.338
Q3 (5.79–6.84)	137/151	0.75 (0.53, 1.06)	0.106	0.72 (0.49, 1.07)	0.105
Q4 (≥6.85)	130/157	0.70 (0.50, 0.98)	0.037	0.67 (0.46, 0.99)	0.046
Categories					
Low (<5.78, median)	307/266	Ref.		Ref.	
High (≥5.78, median)	267/308	0.76 (0.60, 0.97)	0.026	0.75 (0.57, 0.99)	0.039

Note: Models were adjusted for age, ALT, ALB, BMI, sex, smoking status, alcohol drinking, SBP, TC, TG, UA, HDL-C, glucose, creatinine, sleep quality, meat consumption frequency, type of meat consumed, TMAO, oral antihypertensive medication, and family history of cancer.

**Table 3:** The association of L-carnitine with digestive system and non-digestive system cancer risk.

L-Carnitine (μg/mL)	Digestive System			Non-Digestive System		
	CASES/CONTROLS	OR (95% CI)	*p*-Value	Cases/Controls	OR (95% CI)	*p*-Value
Per 1-unit increment	214/214	0.82 (0.69, 0.97)	0.024	361/361	0.87 (0.78, 0.98)	0.016
Quartiles						
Q1	60/46	Ref.		98/83	Ref.	
Q2	59/48	0.88 (0.44, 1.75)	0.633	92/89	0.83 (0.51, 1.34)	0.440
Q3	50/56	0.58 (0.28, 1.22)	0.152	88/92	0.78 (0.48, 1.28)	0.329
Q4	44/63	0.48 (0.23, 0.99)	0.049	83/97	0.61 (0.38, 0.99)	0.043
Categories						
Low (<median *)	119/94	Ref.		190/172	Ref.	
High (≥median *)	94/119	0.57 (0.34, 0.97)	0.038	171/189	0.81 (0.58, 1.13)	0.211

Note: Models were adjusted for age, ALT, ALB, BMI, sex, smoking status, alcohol drinking, SBP, TC, TG, UA, HDL-C, glucose, creatinine, sleep quality, meat consumption frequency, type of meat consumed, TMAO, oral antihypertensive medication usage, and family history of cancer. * The median values of LC for digestive system and non-digestive system cancers were 5.95 and 5.73, respectively.

**Table 4:** The association of TMAO with overall cancer risk.

TMAO (μg/mL)	Cases/Controls (Ratio 1:1)	Crude Model		Adjusted Model	
		OR (95% CI)	*p*-Value	OR (95% CI)	*p*-Value
Continuous (1-unit increment)	574/574	1.07 (0.65, 1.76)	0.801	1.02 (0.57, 1.83)	0.950
Quartiles					
Q1 (<0.10)		Ref.		Ref.	
Q2 (0.10–0.15)		0.84 (0.61, 1.17)	0.300	0.80 (0.55, 1.17)	0.249
Q3 (0.16–0.25)		0.71 (0.50, 1.03)	0.070	0.57 (0.37, 0.90)	0.016
Q4 (≥0.26)		0.91 (0.64, 1.29)	0.598	0.75 (0.49, 1.14)	0.176
*p* for trend			0.285		0.173
Categories					
Low (<median *)		Ref.		Ref.	
High (≥median *)		0.89 (0.69, 1.16)	0.391	0.77 (0.55, 1.04)	0.085

Note: Models were adjusted for age, ALT, ALB, BMI, sex, smoking status, alcohol drinking, SBP, TC, TG, UA, HDL-C, glucose, creatinine, sleep quality, meat consumption frequency, type of meat consumed, LC, oral antihypertensive medication usage, and family history of cancer. Statistically significant values are shown in bold with all *p* values < 0.05. * The median value of TMAO was 0.16.

## Data Availability

Data will be made available upon reasonable request.

## Supplementary Materials

The following are available online at https://www.mdpi.com/article/10.3390/nu14234999/s1, Figure S1: The levels of LC and TMAO in different meat intake frequency groups. Note: ** Statistically significant by SNK method. Mean values were presented.
