# Supplementary figures and images for: The Association of Serum L-Carnitine Concentrations with the Risk of Cancer in Chinese Adults with Hypertension
Source: Nutrients. 2022 Nov 24;14(23):4999. doi: 10.3390/nu14234999 (PMC9738465; doi:10.3390/nu14234999)

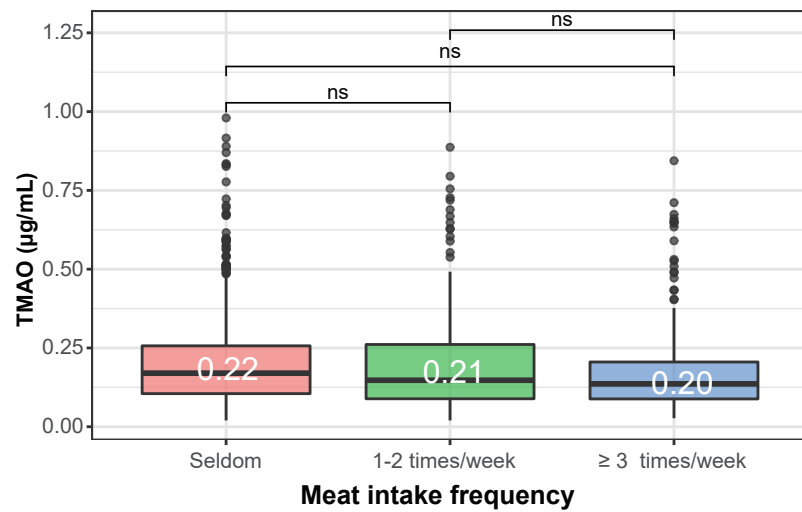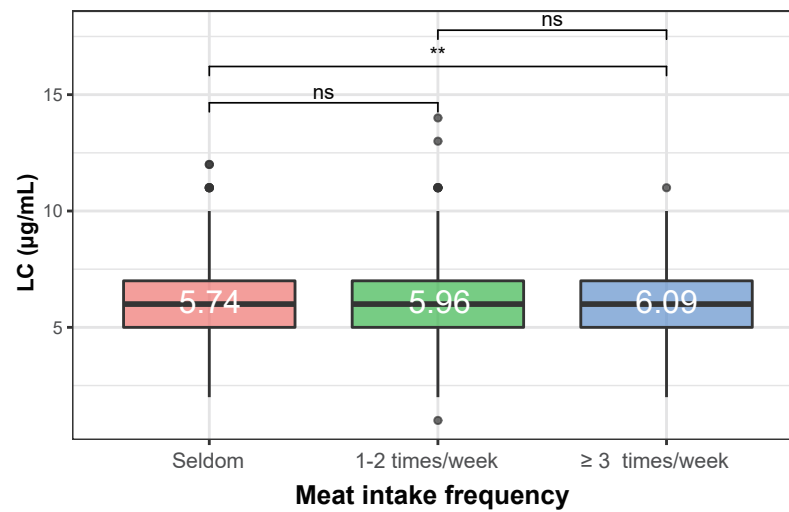

Supplement: Supplementary file 1 [file nutrients-14-04999-s001.zip › nutrients-1959427-supplementary.pdf]
